# The course of diaphragm atrophy in ventilated patients assessed with ultrasound: a longitudinal cohort study

**DOI:** 10.1186/s13054-015-1141-0

**Published:** 2015-12-07

**Authors:** Tom Schepens, Walter Verbrugghe, Karolien Dams, Bob Corthouts, Paul M. Parizel, Philippe G. Jorens

**Affiliations:** Department of Anesthesiology and Critical Care Medicine, Antwerp University Hospital, University of Antwerp, Edegem, Belgium; Department of Critical Care Medicine, Antwerp University Hospital and University of Antwerp, Edegem, Belgium; Department of Radiology, Antwerp University Hospital and University of Antwerp, Edegem, Belgium

## Abstract

**Introduction:**

Mechanical ventilation and the effect of respiratory muscle unloading on the diaphragm cause ventilator-induced diaphragmatic dysfunction (VIDD). Atrophy of the diaphragmatic muscle is a major part of VIDD, and has a rapid onset in most animal models. We wanted to assess the clinical evolution and risk factors for VIDD in an adult intensive care unit (ICU) by measuring diaphragm thickness using ultrasound.

**Method:**

We performed a single-centre observational cohort study, including 54 mechanically ventilated patients. The right hemidiaphragm was measured daily at the zone of apposition on the midaxillary line.

**Results:**

Mean baseline thickness was 1.9 mm (SD ± 0.4 mm), and mean nadir was 1.3 mm (SD ± 0.4 mm), corresponding with a mean change in thickness of 32 % (95 % CI 27–37 %). Length of mechanical ventilation (MV) was associated with the degree of atrophy, whereas other known risk factors for muscle atrophy in an ICU were not. The largest decrease in thickness occurred during the first 72 hours of MV.

**Conclusions:**

Diaphragm atrophy occurs quickly in mechanically ventilated patients and can accurately be monitored using ultrasound. Length of MV, as opposed to other variables, is associated with the degree of atrophy.

**Clinical trial registration:**

Clinicaltrials.gov NCT02299986. Registered 10/11/2014

**Electronic supplementary material:**

The online version of this article (doi:10.1186/s13054-015-1141-0) contains supplementary material, which is available to authorized users.

## Introduction

Muscle weakness and dysfunction are common problems in patients hospitalized in the intensive care unit (ICU) [1–3]. This process affects striated muscles: dysfunction and atrophy are observed, often simultaneously, in muscles of the limbs and of the diaphragm [4, 5]. Whereas general limb muscle wasting is a more gradual and slow process, reaching its peak after the first 2–3 weeks of ICU stay [6, 7], diaphragmatic dysfunction appears to occur much more rapidly [3, 8–10].

Many factors contribute to this intriguing problem in the ICU, but mechanical ventilation (MV) by itself seems to affect the diaphragm. The terminology ‘ventilator-induced diaphragmatic dysfunction’ (VIDD), originally described by Vassilakopoulos and Petrof, was therefore introduced to describe these effects of mechanical ventilation and respiratory muscle unloading on the diaphragm [11, 12]. Apart from inactivity by mechanical ventilation, other factors including inflammation, malnutrition, the use of certain pharmacological agents, and the existence of neuromuscular syndromes prior to ICU admission have been reported to influence diaphragm dysfunction as well [13]. Diaphragmatic dysfunction refers to altered force and structure, and both can be studied [6, 14, 15].

The complex disease process of VIDD represents more than atrophy alone. The dysfunction itself originates at the level of the muscle cell membrane and/or the contractile apparatus, rather than that of the axonal phrenic nerve or the neuromuscular junction [5, 16]. Diaphragmatic muscle thinning is an essential part of VIDD [6, 12].

Functional loss (i.e. the decrease in the diaphragm’s contribution in generating a breath) can be calculated by the Gilbert index [8, 13]. In addition to exploring the loss of contractility, imaging techniques have been used to visualize the excursion and anatomy of the diaphragm. Ultrasonography is becoming increasingly popular in the day-to-day management of ICU patients [6, 11, 14, 15]. It is a simple, non-invasive and safe imaging technique that can be used for the assessment of distinctive diaphragmatic characteristics [17]. These include movement parameters such as amplitude and velocity of contraction, which can be assessed using M-mode ultrasound [18]. In addition, static and dynamic (thickening fraction during inspiration) diaphragmatic thickness can also be measured by ultrasonography [19].

In a small cohort (n = 7) of mechanically ventilated patients, Grosu and colleagues originally demonstrated that the thickness of the diaphragm decreases about 6 % per day [9]. Others have reconfirmed the feasibility of diaphragm thickness recording in ventilated patients [20]. Good reproducibility and repeatability of right hemidiaphragm thickness measurements was demonstrated. Diaphragm thickening, defined as recording the difference between inspiration and expiration in B- or M-mode, could serve as a novel parameter to predict weaning and extubation success [21, 22]. To study both the extent and the time-course of diaphragmatic atrophy, we longitudinally measured end-expiratory diaphragm thickness in a cohort of mechanically ventilated ICU patients.

## Methods

**Fig. 1:**
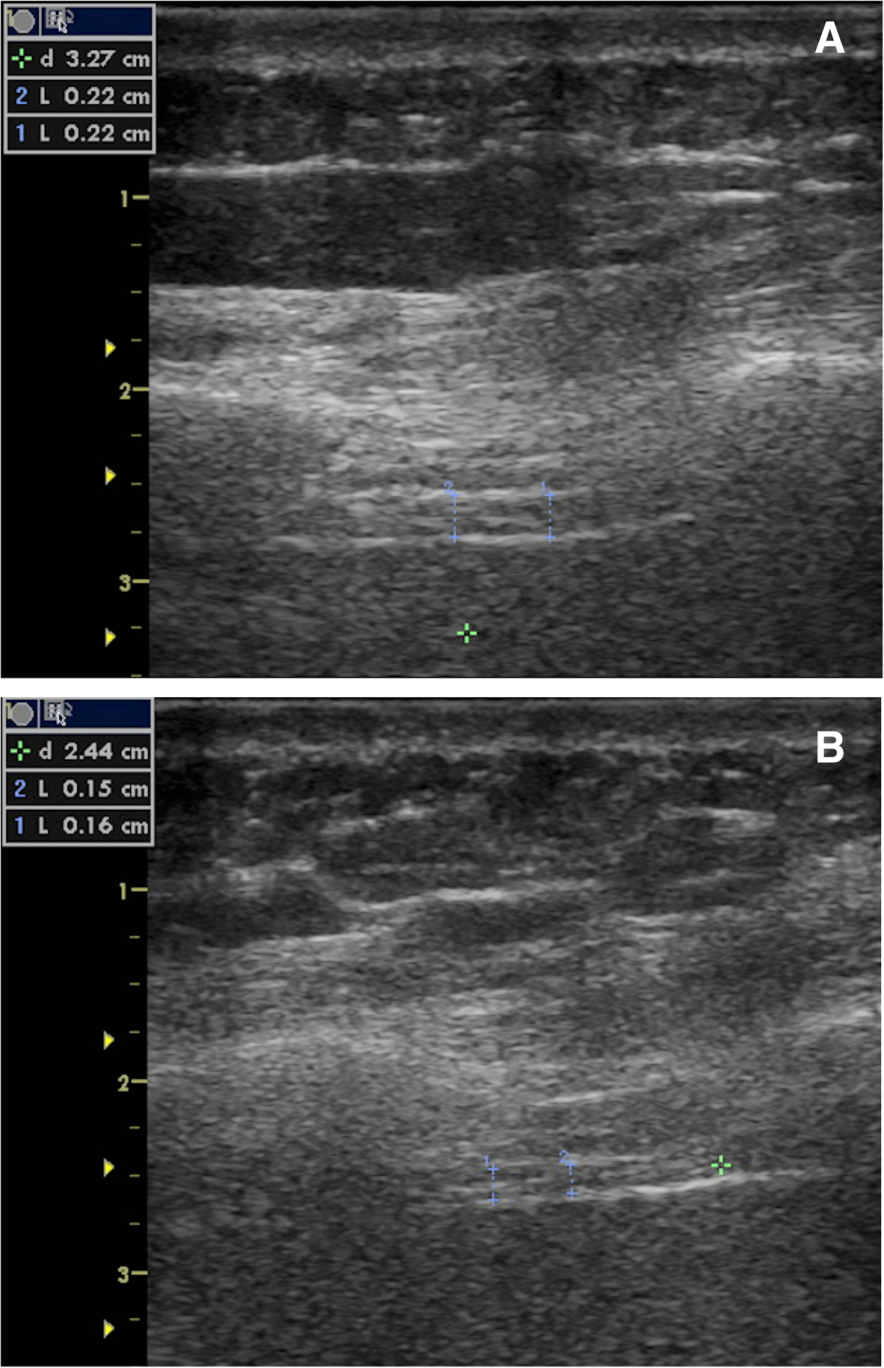
Ultrasound sample images. Right hemidiaphragm thickness recording image after admission (**a**) and on day 5 (**b**) as obtained during the study

This longitudinal, single-centre, observational cohort study was approved by the institutional review board and ethics committee of the Antwerp University Hospital, Antwerp, Belgium (study identifier: 13/06/70), and was registered at Clinicaltrials.gov (NCT02299986). All subjects’ healthcare proxy provided written informed consent. The study was performed in accordance with the ethical standards of the Declaration of Helsinki. Written informed consent was obtained to publish Fig. 1a and b.

### Subjects and data recording

Between May 2013 and June 2015, patients admitted to the intensive care unit (ICU) were evaluated for enrolment into the study. In 56 newly intubated patients, baseline thickness measurements were made within 24 hours after the start of mechanical ventilation (MV). Selection of patients was based on the likelihood of prolonged (>72 h) MV. Exclusion criteria were age <18 years, history of neuromuscular disease or known anatomical malformation of the diaphragm, use of non-invasive ventilation before the start of MV, hemodynamic instability, the presence of a tracheostomy and an admission to an ICU within 12 months prior to this inclusion.

The first ultrasonographic measurement was performed within 24 hours after the start of MV, and the subsequent recordings were acquired daily within a 24 hours ± 4 hours time frame. The same investigator (TS, ICU physician) performed all the recordings. A board-certified radiologist experienced in ultrasonography visually inspected the B-mode still images of the diaphragm and the position of the ‘caliper’ markers as recorded by TS. Recordings were discontinued at the moment of unassisted spontaneous ventilation, tracheostomy, extubation, or when a patient died. Primary outcome parameter was the change in diaphragm thickness from baseline to nadir.

Demographic variables, including the Simplified Acute Physiology Score II (SAPS II) [23], were extracted from the digital patient data management system (Metavision, iMDsoft, Tel Aviv, Israel). Secondary outcome parameters were patient characteristics associated with a decrease in diaphragm thickness: age, sex, SAPS II, duration of MV, percentage of time in controlled MV modes, use of corticosteroids during ICU stay, sepsis, continued use of neuromuscular blocking agents and aminoglycosides antibiotic use as possible associated risk factors, as they are known risk factors for ICU-acquired weakness [10, 14, 24–26]. All patients were sedated in accordance with our sedation protocol. Briefly, patients were sedated with propofol plus remifentanil drips, titrated to a Richmond Agitation-Sedation Scale set by the treating ICU physician. If these drugs were insufficient in sedating the patient adequately, midazolam could be added. Neuromuscular blocking agents usage is left to the discretion of the treating ICU physician, and was never routinely administered in our patients.

### Measurement technique

Diaphragm thickness was measured ultrasonographically, using a standardized technique at the zone of apposition on the midaxillary line. This was first described in 1997 by Cohn et al., and since then applied by others [9, 20, 27, 28]. As in the study by Goligher et al. [20], it proved difficult to consistently visualize the left hemidiaphragm, so measurements of left hemidiaphragm thickness were not performed in this study. We recorded the type of applied ventilation mode hourly, as this may have its effect on the evolution of diaphragm thickness, and included the amount of time in controlled ventilation modes as a possible predictor for diaphragm atrophy. More details about the measurement technique and the recorded ventilator parameters are noted in Additional file 1.

Before commencing the study, both intra- and inter-observer variability of diaphragm ultrasound recordings in both ventilated patients and non-ventilated volunteers on different time points were performed, by TS and with a board-certified radiologist experienced in ultrasonography (BC). The coefficients of reproducibility (Guttman) for inter-observer variability for TS were 0.875 for the volunteers study and 0.971 for the ventilated patients study. The coefficients of reproducibility for intra-observer variability for TS were 0.899 for the volunteers study and 0.945 for the ventilated patients study.

### Statistics

Statistical analyses were performed with SPSS Statistics software, version 20.0 for Mac (IBM Corp., Armonk, NY, USA) Results were reported as mean ± standard deviation (SD) or median (interquartile range (IQR)). The relationships between patient characteristics (including the risk factors) and diaphragm thickness changes were assessed using the Mann-Whitney *U* test and linear regression models. Changes in baseline and nadir diaphragm thickness were analysed using the Wilcoxon signed-rank test. All analysed risk factors for atrophy were entered in a multivariate regression model. The level of statistical significance was set at 0.05. The statistical methods are explained in detail in Additional file 1.

## Results

### General characteristics

Fifty-six mechanically ventilated patients were enrolled, of which 54 patients were ventilated for at least 72 hours and thus provided sufficient data to be analysed. In all analysed patients the right hemidiaphragm was measured. One patient who had a chest drain inserted in the right pleural space was excluded for further analysis because no reliable measurements could be performed on the right hemidiaphragm, totalling the number of subjects that could be analysed to 53. We were able to successfully record the diaphragm thickness in 100 % of the attempts for all patients, with a total of 308 measurements. The maximal change in diaphragm thickness (from baseline to nadir) was calculated for each patient.

Clinical characteristics of the included patients are shown in Table 1. Median SAPS II score was 72 (IQR 63–80). Median time on the ventilator was 8 days (IQR 4–14 days).

**Table 1:** Patient characteristics and outcome parameters

Age (median, IQR)	67 (55-75)
Male/female	33/20
SAPS II (median, IQR)	72 (63-80)
ICU mortality (n, %)	20 (38)
Maximal change in thickness (mean %, 95 % CI)	32 (27-37)
Aminoglycoside (AG) use (n, %)	15 (28)
Maximal change in thickness in AG subgroup (mean %, SD)	38 (18)
Maximal change in thickness in non-AG subgroup (mean %, SD)	30 (18)
*P*	0.367
Corticosteroids (CS) use (n, %)	26 (49)
Maximal change in thickness in CS subgroup (mean %, SD)	34 (17)
Maximal change in thickness in non-CS subgroup (mean %, SD)	29 (19)
*P*	0.160
Continuous neuromuscular blocking agents administration (n, %)	14 (28)
Single dose or more of neuromuscular blocking agents administration (n, %)	35 (66)
Sepsis on admission (n, %)	25 (47)
Maximal change in thickness in sepsis subgroup (%, SD)	35 (28-42)
Sepsis during ICU stay (n, %)	45 (85)

*IQR* interquartile range, *SAPS II* Simplified Acute Physiology Score II, *ICU* intensive care unit, *CI* confidence interval, *SD* standard deviation

The first measurement was performed within 24 hours after initiation of ventilation (mean 13 h and 08 min, SD 7 h 50 min). In total, 14 patients received continuous muscle relaxant administration (cisatracurium) at any given moment while receiving MV. Furthermore, 35 patients in total received one or more bolus administrations of a muscle relaxant (either rocuronium of cisatracurium).

### Diaphragmatic thickness in ventilated patients over time

Mean baseline thickness was 1.9 mm (SD ± 0.4 mm). Compared to baseline thickness, the last recorded diaphragm thickness before the moment of ventilator liberation, tracheotomy, death, or end of assisted ventilation in the intubated (brain) dead patient was decreased by more than 10 % in the vast majority of subjects (n = 40, 77 %), remained stable in ten (19 %) and increased by more than 10 % in two (4 %) subjects. The rate of diaphragm thickness decline was 10.9 % per day on average (SD 10.2 %).

**Fig. 2:**
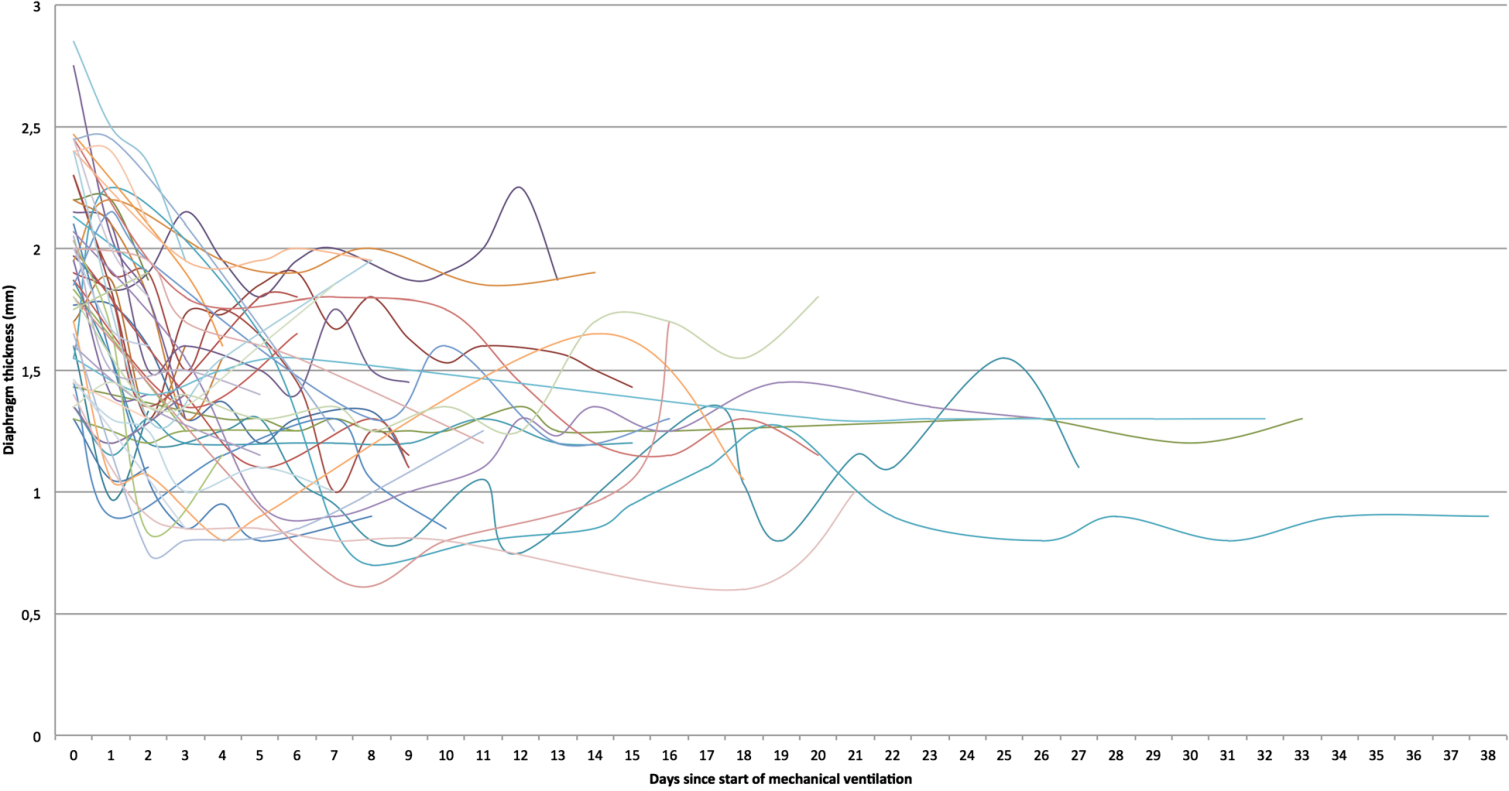
Diaphragm thickness measurements. Raw values of all diaphragm thickness recordings in millimetres for each day of mechanical ventilation. Lines use a Bézier curve construction (see Additional file 1)

**Fig. 3:**
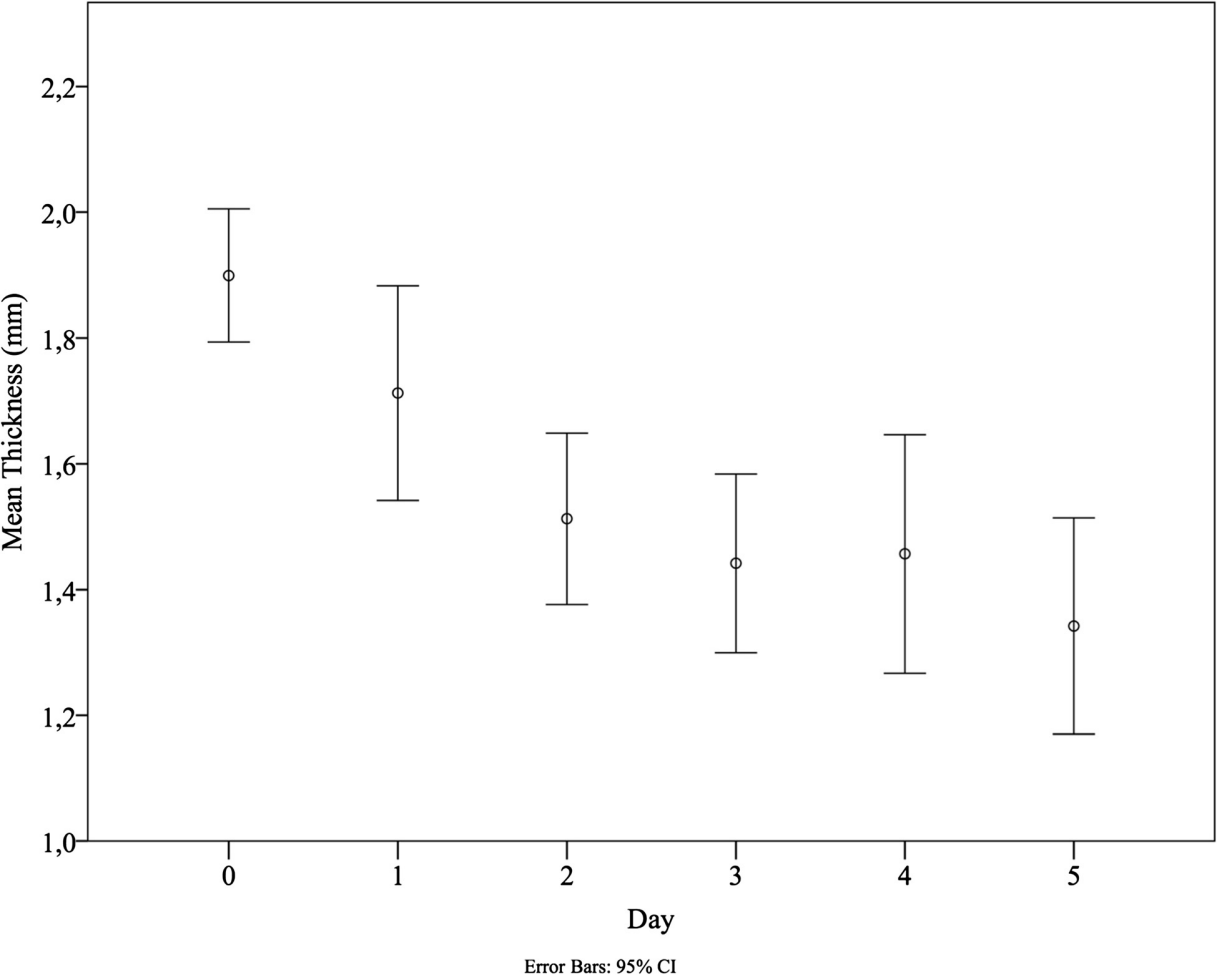
Diaphragm thickness measurements. Mean thickness of the diaphragm with 95 % CI as recorded with ultrasound from baseline recordings (day 0) to 5 days after baseline

Mean nadir was 1.3 mm (SD ± 0.4 mm), corresponding with a mean maximal decrease in thickness of 32 % (95 % CI 27–37 %) for all patients combined. After 24 hours of ventilation, we already see a drop of diaphragm thickness of 9 % (mean thickness 1.7 mm, SD ± 0.5 mm, *P* = 0.008 versus baseline). After 48 and 72 hours of ventilation, diaphragm thickness decreases with respectively 20 % (mean thickness 1.5 mm, SD ± 0.4 mm) and 26 % (mean thickness 1.4 mm, SD ± 0.4 mm) versus baseline recordings, illustrating the rapid progression of the atrophy in VIDD. Median time to nadir was 3 days (IQR 2–7 days). The change in thickness after 72 hours did not correlate with the time spent on the ventilator afterwards. Fig. 2 shows all diaphragm thickness recordings over time for all included patients. Fig. 3 shows the mean thickness of the diaphragm from baseline to day 5.

### Risk factors associated with diaphragm thinning

Length of ventilation was associated with diaphragm thinning (*F* = 4.7, *P* = 0.034). Mean time in controlled ventilation modes was 58 % (SD 28 %). Mean time spent in controlled ventilation modes from start of MV to nadir was 77 % (SD 42 %). Age, gender, disease severity (SAPS II) on admission, steroid use during ICU stay, the administration of single or continued doses of muscle relaxants, or sepsis on admission were not associated with maximal decrease in diaphragm thickness. The overall time in assisted versus controlled ventilation modes was not associated with the degree of atrophy, and neither was the time in assisted versus controlled ventilation modes from start of MV to nadir. The percentage of time in controlled MV, sepsis status, corticosteroids administration and aminoglycoside administration parameters were entered in a multivariate regression model, but none of these parameters reached significance (full results in Additional file 1). A subgroup analysis was performed for the sepsis patients who were diagnosed with sepsis upon admission. Mean change in thickness was not faster in the sepsis group compared to the non-sepsis group, when considering decrease in thickness 24, 48 or 72 hours after the start of MV.

## Discussion

In this study, we report a longitudinal follow-up of diaphragm thickness in a number of mechanically ventilated patients in the ICU. We demonstrate a rapid progression of diaphragm atrophy, with already a significant decrease in thickness after just 1 day of MV, and the largest decrease in diaphragm thickness occurring during the first 72 hours of MV. The degree of atrophy in our cohort is associated with the length of ventilation, but not with other known risk factors for muscle atrophy, including sepsis or corticosteroid use.

Diaphragm muscle atrophy in ventilated patients was previously demonstrated in humans: as demonstrated in muscle biopsies in brain-dead organ donors [3, 6]: Jaber and co-workers found a 39 % difference in mean cross-sectional area between short- (<6 h) and long-term ventilated patients [3], and the difference was even greater in the study by Levine et al., with a difference of 57 % and 53 % in cross-sectional areas of slow-twitch and fast-twitch fibres respectively versus biopsies in short-term (2–3 h) ventilated patients [6]. Furthermore, in this study, as little as 18 hours of controlled MV resulted in noticeable atrophy of diaphragm muscle fibres. Biopsy, as a way of evaluating atrophy is hindered by the impossibility to make repeated measures, and thus unavoidably comparing the thickness in two different groups.

In our study, the thickness of the diaphragm was measured by a validated ultrasound technique [27, 28]. Grosu and co-workers were the first to report the use of repeated ultrasound recordings in seven ventilated patients to assess diaphragm atrophy [9]: a 6 % decrease in diaphragm thickness per day of MV was shown. Our study shows a comparable result, with a decrease of 9 % after one day of MV, and a total decrease of 21 % and 26 % after 2 or 3 days of MV respectively. These findings illustrate that diaphragm atrophy is a rapidly evolving process, with an exponential decline in thickness.

A logarithmic relationship between length of ventilation and diaphragmatic strength was already demonstrated by Hermans et al. using twitch transdiaphragmatic diaphragmatic pressure generation recordings [14]. Jaber et al. performed a similar investigation and demonstrated a mean decrease of 32 % in diaphragm force in long-term ventilated patients [3]. Furthermore, they were able to associate diaphragm atrophy with a decrease in twitch transdiaphragmatic diaphragmatic pressure generation. Although the results are interesting, technical difficulties are likely to preclude this technology from being an easy-to-use bedside tool for longitudinal diaphragm function follow-up.

In septic ICU patients, atrophy is more pronounced in the diaphragm compared to limb muscle [29]. In our cohort, we could not associate diaphragm atrophy with sepsis [30], but this does not exclude that inflammation may have had effects on contractile dysfunction without associated additional atrophy, which we did not measure. The ultrastructural injury may be more pronounced in the sepsis subgroup, and thereby resulting in potentially more pronounced degrees of VIDD [10].

Inactivity of the diaphragm is present in patients that are on partially assisted or controlled mechanical ventilation [20]. In our cohort, the diaphragm muscle atrophy was heterogeneously present, and could not be directly associated with the amount of time the patient was ventilated in a controlled or assisted mode of ventilation. There is, however, no guarantee that a diaphragm is active during assisted modes of ventilation, for patients can trigger the ventilator using secondary respiratory muscles [31]. Interestingly, in the study by Hermans et al., longer periods of support ventilation or bi-level positive-pressure ventilation were also linked to increased diaphragm dysfunction [14]. Furthermore, rats exposed to controlled MV or breathing spontaneously during propofol sedation for 24 hours had a similar degree of diaphragm atrophy, suggesting that diaphragm activity may not always be protective for VIDD [32]. Whether maintaining diaphragm activity during MV is protective for VIDD or atrophy in humans remains to be answered [33]. Diaphragm pacing was successfully tested as a preventive strategy in ventilated sheep [34], and intermittent spontaneous breathing has been reported as a protective strategy in a rat model [35]. It does seem to be a plausible solution, because mechanical inactivity of muscles leads to an oversupply of reactive oxygen species (ROS) in dysfunctional mitochondria, triggering the VIDD cascade [36].

Our findings are in accordance with the data described in a very recent manuscript by Goligher and co-workers [37]. Diaphragm thickness and contractile activity (quantified by the inspiratory thickening fraction) in their group of ventilated patients decreased by 10 % or more in 44 % of all cases, remained unchanged in 44 %, and even increased by more than 10 % in a minority (12 %). In our study group, diaphragm thickness decreased more than it did in Goligher’s cohort. Diaphragm thickness decreased by more than 10 % in 40 subjects (77 %), remained stable in ten (19 %) and increased by more than 10 % in two (4 %) subjects. It would be interesting to know the exact relationship between the evolution of diaphragmatic thickness and the duration of weaning or weaning effort. Unfortunately, as our ICU habits and protocol allow great variability of weaning strategies between subjects we could not really explore the relationship as the exact time when weaning ‘started’ cannot be taken into account. As the recent data in Goligher’s study [37] showed that contractile activity of the diaphragm decreased with increasing ventilator driving pressure (*P* = 0.01) and controlled ventilator modes (*P* = 0.02), one might assume that titrating ventilatory support to maintain normal levels of inspiratory effort may prevent changes in diaphragm configuration associated with mechanical ventilation. For the time being, awaiting results from newer studies taking the weaning process into account, we can only hypothesize that diaphragm thinning is a feature of diaphragm dysfunction with associated worse clinical respiratory outcome.

Other therapeutic strategies, apart from the possibility of continued activity, remain to be explored as well. Regretfully, up to now, we do not have a clear treatment plan for patients with VIDD available. Further trials are necessary to determine whether MV can evolve from lung protective towards a muscle protective MV. Given the quick onset of VIDD in ventilated patients, it seems prudent to assume that muscle protection may be needed as early as possible. The diaphragm appears to be exceptionally susceptible for disuse atrophy, more so than limb muscles, and even more when sepsis is present [29].

Unavoidably, our study has several limitations. We measured a morphological characteristic, and atrophy is not necessarily linked with muscle strength. In this study, we only evaluated thickness and not diaphragm function or strength.

However, data demonstrate the association between atrophy and muscle strength in VIDD [3]. A second limitation is that inflammation and lipid overload may cause swelling of muscle cells, and affect thickness recordings [36]. A third limitation is that we do not have a real baseline thickness recording for our patients. All baseline thickness measurements were performed after the start of mechanical ventilation, albeit within a few hours after the initiation. Whether decline of diaphragmatic thickness occurs in ICU patients regardless of mechanical ventilator support needs to be determined too.

## Conclusions

This study describes the day-by-day evolution of diaphragm atrophy in VIDD in ICU patients, measured by ultrasonography. Mean baseline thickness was 1.9 mm and mean nadir was 1.3 mm corresponding with a mean change in thickness of 32 %. Length of MV, as opposed to other known risk factors for muscle atrophy, was associated with the degree of diaphragm atrophy. The diaphragm atrophy occurred fast, with the largest decrease in thickness occurring during the first 72 hours of MV. Further research is needed to determine whether MV can evolve from lung protective towards a muscle protective MV.

## Key messages

- Diaphragm atrophy in ventilated patients occurs fast
- Diaphragm thickness can be monitored using ultrasound to assess the daily evolution and the effect of different ventilation strategies
- Strategies to prevent diaphragm muscle loss should be applied early in the course of mechanical ventilation

## Additional file

Additional file 1:
**Supplemental details of Measurements, Statistics and Multivariate analysis.** (DOCX 32 kb)
