# Supplementary material for: The course of diaphragm atrophy in ventilated patients assessed with ultrasound: a longitudinal cohort study
Source: Crit Care. 2015 Dec 7;19:422. doi: 10.1186/s13054-015-1141-0 (PMC4671211; doi:10.1186/s13054-015-1141-0)
Supplement: Additional file 1: — Supplemental details of Measurements, Statistics and Multivariate analysis. (DOCX 32 kb) [file 13054_2015_1141_MOESM1_ESM.docx]

**Additional file 1: Methods and results**

Measurement technique

A linear 13 MHz ultrasound transducer probe (GE Vivid-I 12L-RS) was used for all patients in two-dimensional B-mode; the probe was positioned in the eighth or ninth intercostal space on the midaxillary line (zone of apposition) and angled perpendicular to the chest wall. Subjects were reclining in bed at a 30° - 45° angle.

The right diaphragm was identified as a three-layered structure just above the liver, appearing as a hypoechogenic muscular layer bounded by echogenic membranes of pleura. The diaphragm was further identified dynamically as the most superficial structure that was obliterated by the leading edge of the lung upon inspiration. Images were obtained at end-expiration[9].

Using this technique, we measured diaphragm thickness, defined as the distance from the outside of the pleura to the outside of the peritoneum, to the nearest 0.1 mm and recorded a digital image.

Ventilation modes and parameters

Patients could be ventilated in assisted, controlled or hybrid ventilation modes. A hybrid ventilation mode is a controlled ventilation mode with the added opportunity for the patient to receive a supported breath if he or she triggers the ventilator, the so called Assist/Control (A/C) modes and Synchronized Intermittent Mandatory Ventilation (SIMV) modes. Hybrid ventilation modes were added to the group of controlled ventilation modes when calculation the time spent in controlled ventilation modes.

The ventilator settings and extrinsic positive end-expiratory pressure (PEEP) levels were recorded upon inclusion and then daily. PEEP level was set to 5cm H_2_O at the moment of the daily measurement. Measuring the diaphragm thickness for each patient was done by observing real-time graphics of airflow and airway pressure at the point of end-expiration to assure that there was no change in ventilator parameters, such as intrinsic PEEP, which might affect lung volumes and hence thickness.

Statistical methods

Normality was assessed using the Kolmogorov-Smirnov test and results were reported as mean ± standard deviation (SD) for normally distributed or median (interquartile range) for non-normally distributed data.

The categorical independent variables that were included in the analysis are: sex, use of corticosteroids during intensive care unit (ICU) stay, sepsis on admission, continued use of neuromuscular blocking agents and use of aminoglycoside antibiotics. Association of these individual parameters with nadir change in diaphragm thickness was assessed using a Mann-Whitney U test.

The continuous independent variables that were included are: the Severe Acute Physiology Score II (SAPS-II), duration of mechanical ventilation (MV), and percentage of time in controlled MV. Association of these individual parameters with nadir change in diaphragm thickness was assessed using a linear regression model.

Changes in baseline and nadir diaphragm thickness were analyzed using the Wilcoxon signed rank test. The percentage of time in controlled MV, sepsis status, corticosteroids administration and aminoglycoside antibiotic administration parameters were entered in a multivariate linear regression model with nadir change in diaphragm thickness as the dependent variable. A subgroup analysis was performed for the patients who were diagnosed with sepsis upon admission. Decrease in thickness 24, 48 or 72 hours after the start of MV was compared between the sepsis and non-sepsis group using the Student’s T-Test. We calculated the % of time in controlled ventilation modes from start to nadir and examined this for a possible association with nadir change in thickness using a linear regression model. Correlation between change in thickness after 72 hours with the spent on the ventilator afterwards was also examined using a linear regression model.

Figure 1

The lines in figure 1 are constructed with a Bézier algorithm, which is routinely used by Microsoft® Excel®. The Bézier curve construction adds a smoothed visual effect to the curves, but guarantees that the line passes through all the individual recorded values.

Multivariate regression model

Nadir change in diaphragm thickness as the dependent variable:

| Parameter | B (unstandardized) | *P* |
| --- | --- | --- |
| Corticosteroid administration | 1.920 | 0.730 |
| Percentage of time in controlled MV | 0.061 | 0.513 |
| Aminoglycoside administration | 9.245 | 0.106 |
| Sepsis on admission | 3.922 | 0.468 |
